# CERES forms a complex with RH37 and eIF4E

**DOI:** 10.1007/s00299-026-03888-5

**Published:** 2026-08-26

**Authors:** Isabel C. Okeke, Alfonso Muñoz, M. Mar Castellano

**Affiliations:** 1 Centro de Biotecnología y Genómica de Plantas, Universidad Politécnica de Madrid (UPM) — Instituto Nacional de Investigación y Tecnología Agraria y Alimentaria (INIA/CSIC), Campus de Montegancedo (UPM), Pozuelo de Alarcón, 28223 Madrid, Spain; 2 Departamento de Sistemas y Recursos Naturales, ETSI de Montes, Forestal y del Medio Natural, Universidad Politécnica de Madrid, 28040 Madrid, Spain

**Keywords:** CERES, EIF4E1, Translation initiation, DEAD-box helicases, Ded1, DDX3, Protein synthesis

## Abstract

**Key message:**

The translation initiator CERES interacts with the RNA helicase RH37 and bridges the interaction of RH37 with eIF4E.

**Supplementary Information:**

The online version contains supplementary material available at https://doi.org/10.1007/s00299-026-03888-5.

## Introduction

Plants deploy specialized transcriptional and post-transcriptional programs during their growth and development. Among these, translation plays a crucial role, as it enables plants to rapidly adjust protein production and mount timely responses to external stimuli (Castellano and Merchante 2021).

CERES is a plant-specific leucine-rich repeat (LRR) protein involved in translation regulation. In Arabidopsis, it interacts with the eIF4E isoforms and forms complexes with additional translation initiation factors. These complexes, through a noncanonical initiation mechanism, recruit the translational initiation machinery and promote protein synthesis at specific stages of the diel cycle (Toribio et al 2019). Beyond its role in global translation, Ribo-seq analyses showed that CERES selectively modulates the translation of mRNAs associated with light and carbohydrate metabolism (Toribio et al 2019). However, the mechanism by which CERES complexes achieve this specificity remains unknown. CERES has also been implicated in dsRNA-induced pattern-triggered immunity (PTI), although the underlying mechanisms and whether they are translation-dependent remain unresolved (Chi et al 2026).

DEAD-box RNA helicases constitute the most abundant helicase family in eukaryotes (Li et al 2023). Within this group, the Ded1/DDX3 subfamily has been implicated in multiple aspects of RNA metabolism in yeast and mammals (Ryan and Schröder 2022). These proteins also play central roles in canonical translation initiation, partly through their interaction with eIF4E (Sharma and Jankowsky 2014). In plants, although less extensively characterized, Ded1/DDX3 homologs display both conserved and plant-specific functions, including the regulation of translation initiation during immune responses (Xiang et al. 2023). Nevertheless, how RNA helicases contribute to these translation-related processes remains unclear.

## Results and discussion

### CERES interacts with the Ded1/DDX3 RNA helicase RH37 in vivo

**Fig. 1:**
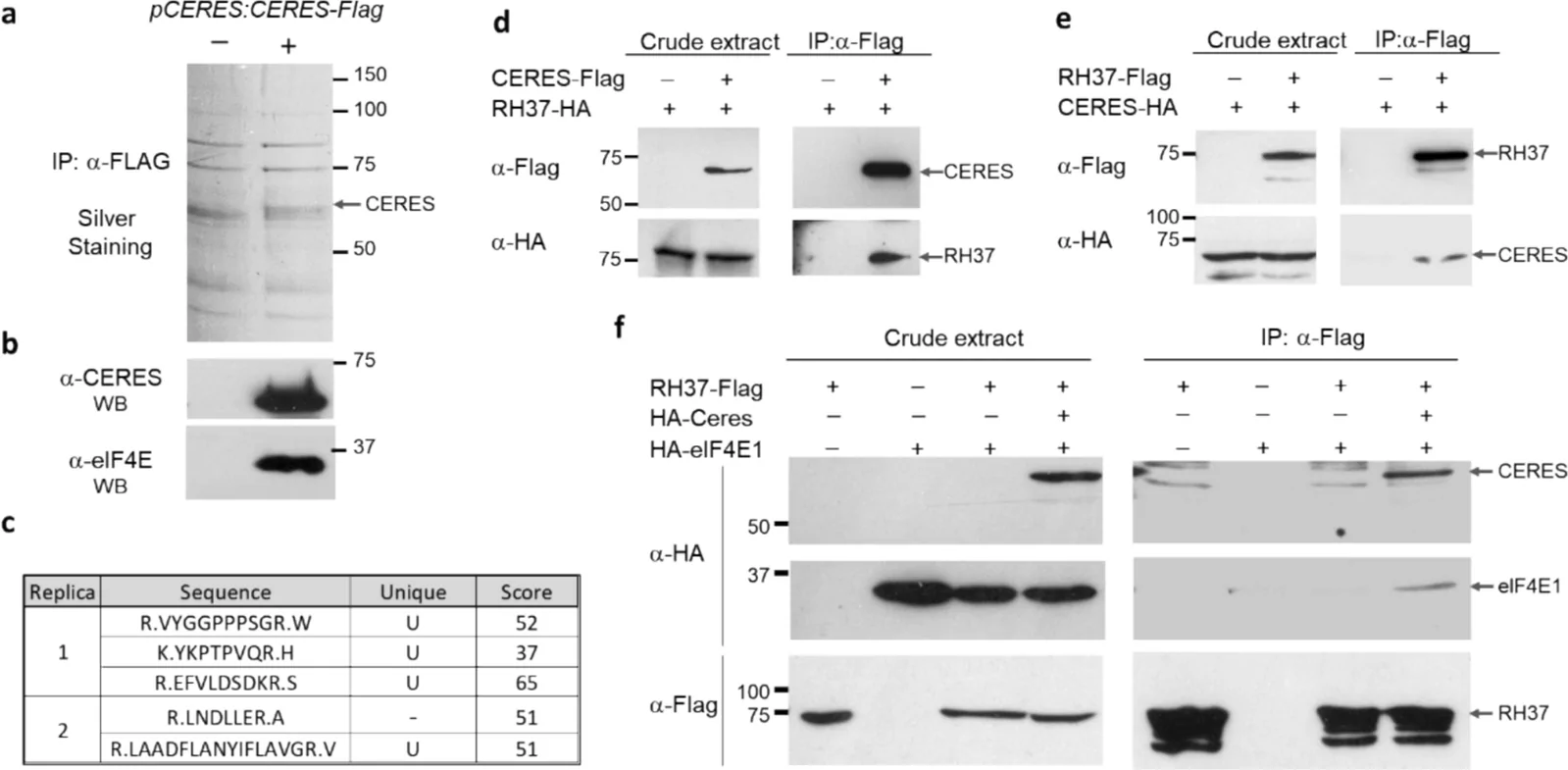
CERES interacts with RH37 in planta and the presence of CERES facilitates the assembly of a RH37-CERES-eIF4E1 complex in vivo. **a** Co-immunoprecipitation of CERES interactors. Extracts from either Col-0 (negative control) or *pCERES:CERES-Flag* seedling tissue were immunoprecipitated using the anti-Flag resin. **b** The presence of CERES and eIF4E in the eluted fractions was analyzed by western blot using anti-CERES and anti-eIF4E antibodies. **c** In addition, two different replicates of the eluates from Col-0 (negative control) or *pCERES:CERES-Flag* seedling tissue were analyzed by LC-ESI MS/MS and the identified peptides corresponding to AtRH37 were annotated. The sequence, whether these peptides are unique for AtRH37(U) and the score of the identified RH37 peptides in each replicate are shown. No such peptides were detected in the control samples. **d–f** Directed immunoprecipitation analysis for interaction analysis. Protein extracts (crude extracts) from *N. benthamiana* leaves transiently expressing, under the control of the *35S* promoter, different combinations of (**d**) CERES-Flag and RH37-HA, **e** RH37-Flag and CERES-HA and **f** RH37-Flag and HA-eIF4E1 and HA-CERES were subjected to immunoprecipitation using anti-Flag beads. The presence of the different proteins in the crude extracts and in the eluted fractions from the immunoprecipitations (IP:α-Flag) was analyzed by western blot using anti-HA and anti-Flag antibodies. All co-immunoprecipitation experiments were repeated independently three times with similar results. The numbers at the right or left of the panels correspond to the weight (kDa) of standard protein molecular markers

To identify novel CERES interactors that could shed light on its role in translation regulation in plants, we generated transgenic lines expressing *pCERES:CERES-Flag*. Protein extracts from these lines and from Columbia-0 (Col-0) (the wild-type line used as control) were subjected to CERES immunoprecipitation using anti-Flag resin and the eluates were further tested for the presence of CERES and subjected to MS/MS identification. As observed in the silver staining of the eluted proteins, a band of ⁓67 kDa corresponding to CERES is selectively immunoprecipitated in the *pCERES:CERES-Flag* extracts (Fig. 1a)*.* The presence of this protein in the eluted fractions from the extracts expressing CERES and their absence in the control immunoprecipiates was further validated using antibodies that recognize AtCERES (Fig. 1b, upper panel). As expected from the described data, CERES was selectively identified by MS/MS in the co-immunoprecipitated fractions from *pCERES:CERES-Flag* (with > 30 peptides in each sample, not shown). Importantly, several eIF4E1- and RH37- peptides were consistently detected to co-immunoprecipate with CERES in the *pCERES:CERES-Flag* samples, but not in the control ones (Supplementary Table 1 for eIF4E1 and Fig. 1c for RH37). The specific co-immunoprecipitation of eIF4E with CERES in the initial eluted fractions was also validated by western blot analysis using specific antibodies against plant eIF4E (Fig. 1b, lower panel). Although eIF4E1 served as a positive control due to its previously described interaction with CERES (Toribio et al 2019), RH37 represents a newly identified CERES interacting protein.

The RH37 and CERES interaction in planta was further validated by directed co-immunoprepitation analyses after the expression of tagged-proteins in *Nicotiana benthamiana* leaves. As shown, RH37 co-immunoprecipitated specifically with CERES (Fig. 1d) and CERES co-immunoprecipitated specifically with RH37 (Fig. 1e). No unspecific binding of RH37 or CERES to the Flag resin was observed, confirming the selectivity of the interaction. These assays were carried out in the absence of external RNase or RNase inhibitors, getting a similar result when the co-IP was treated with an RNase treatment (not shown). These results suggest, in two different plant experimental systems, that CERES interacts in vivo with the RH37, a member of the plant DDX3 RNA helicase subfamily.

### RH37 does not efficiently interact with eIF4E1

A characteristic feature of the Ded1/DDX3 subfamily is the presence of a putative eIF4E-binding motif (Y(V/I)PPHLR) in the N-terminal domain, which is also found in AtRH37 (Supplementary Fig. 1a). Interestingly, its homologs DDX3 and Ded1 have been reported to bind eIF4E in mammals and yeast, although the contribution of this motif to the interaction remains under debate (Shih et al 2008; Gulay et al 2020). Based on this information, we examined whether RH37 interacts with eIF4E1 in vivo. To this end, we co-expressed RH37-Flag and HA-eIF4E1 in *N. benthamiana* leaves and performed the immunoprecipitation of RH37. Unexpectedly, we did not detect an efficient co-immunoprecipitation between RH37 and eIF4E1 in vivo (Supplementary Fig. 1b). These results suggest that, unlike their yeast and mammalian counterparts, AtRH37 does not efficiently bind eIF4E on its own.

### CERES promotes the formation of a eIF4E1–CERES-RH37 complex

Our proteomic data indicate that CERES interacts with both eIF4E1 and RH37 and these results could be consistent with CERES interacting independently with each partner or, alternatively, with CERES acting as a bridge facilitating the formation of a ternary complex.

To distinguish between these possibilities, we co-expressed different combinations of HA-tagged CERES and eIF4E1 together with RH37-Flag in *N. benthamiana* leaves and assessed RH37–eIF4E1 interaction in the presence or absence of CERES. As shown in Fig. 1f, and as expected from Supplementary Fig. 1b, eIF4E1 was not efficiently detected in RH37 immunoprecipitations in the absence of CERES. However, when CERES was co-expressed, eIF4E1 co-immunoprecipitated with RH37 along with CERES.

Here, we provide evidence for the in vivo interaction between CERES and RH37, a member of the Ded1/DDX3 RNA helicase subfamily. We further show that, despite the presence of a putative eIF4E-binding domain in RH37, and, in contrast to its homologs in mammals and yeast, this helicase does not seem to efficiently bind eIF4E1 in vivo; however, its interaction with eIF4E1 seems to be enhanced in the presence of CERES. These results suggest that CERES acts as a scaffolding protein, promoting the assembly of a RH37–CERES–eIF4E1 complex. This work provides the first evidence linking CERES to the RH37 RNA helicase and establishes a new conceptual framework for understanding how plant-specific and conserved translational regulators converge into functional complexes. This novel finding may pave the way for elucidating the biological role of the RH37–CERES–eIF4E1 complex, which will likely require the identification of its target mRNAs. These mRNAs may overlap with those already known to be regulated by CERES or by members of the AtDDX3 subfamily. However, given that both CERES and RH37 remain poorly characterized, it is also plausible that this complex regulates additional, yet unidentified mRNAs and/or biological processes.

## Materials and methods

### Constructs and molecular cloning

To obtain the *pCERES:CERES-Flag* construct, a genomic fragment expanding from −1700 to + 3022 bp position of *CERES* gene was cloned into *pGWB10*. The construct *p35S:RH37-Flag* was obtained by cloning the *RH37* CDS in frame with the Flag epitope in *pGWB11* (Parcy CNRS Grenoble). In both cases the cloning was performed using the Gateway cloning system (Life Technologies). The constructs *p35S:HA-(CERES* and *eIF4E1*) were previously described in (Toribio et al 2019).

### Plant material and growing conditions

For the immunoprecipitation followed of LC-ESI MS/MS analyses, *Arabidopsis thaliana* ecotype Columbia-0 (Col-0) was used as wild-type genetic-background and transgenic *pCERES:CERES-Flag* plants were obtained by introducing the cited constructs in Col-0 background using the floral dip method. Seeds were surface sterilized, stratified at 4 °C for 48 h and grown on plates with Murashige and Skoog (MS) medium supplemented with 1% (w/v) sucrose at 22 °C under a 16 h light photoperiod for 7 days.

### Immunoprecipitation analysis and in solution protein digestion and LC-ESI MS/MS

Immunoprecipitation analyses from *N. benthamiana* or Arabidopsis extracts and in solution protein digestion and LC-ESI MS/MS was carried out as described in (Fernandez-Bautista et al. 2018). Western blots of the eluates from Arabidopsis were carried out using anti-CERES and anti-eIF4E antibodies (kindly provided by Dr. J. L. Gallois, INRA, France).

## Supplementary Information

Below is the link to the electronic supplementary material.

Supplementary file1 (DOCX 171 KB)

## Data Availability

Constructs and lines are available upon reasonable request.
