# Supplementary material for: CERES forms a complex with RH37 and eIF4E
Source: Plant Cell Rep. 2026 Aug 26;45(9):267. doi: 10.1007/s00299-026-03888-5 (PMC13518413; doi:10.1007/s00299-026-03888-5)
Supplement: Supplementary file 1 [file 299_2026_3888_MOESM1_ESM.docx]

**Supplementary Table 1**

| Replicate | Protein | Sequence | Unique | Score |
| --- | --- | --- | --- | --- |
| 1 | AteIF4E1 | R.ISIWTK.N | U | 36 |
|  |  | K.WEDPICANGGK.W |  | 55 |
|  |  | [K.NASNEAAQVSIGK.Q](http://peptide_view.pl/?file=E%3A%2FMASCOT%2Fdata%2F20170327%2FF109157.dat&query=10035&hit=1&index=O23252&db_idx=1&px=1&section=5&ave_thresh=30&_ignoreionsscorebelow=-1&report=0&_sigthreshold=0.05&_msresflags=1097&_msresflags2=258&percolate=-1&percolate_rt=0&_minpeplen=5&sessionID=all_secdisabledsession) | U | 105 |
|  |  | K.LAHGADFYCFK.H |  | 71 |
|  |  | K.SVVTEEAKPNSIENPIDR.Y | U | 107 |
|  |  | K.EFLDYNNSIGFIIHEDAK.K | U | 104 |
|  |  | K.SGVPESHPLEHSWTFWFDNPAVK.S | U | 44 |
|  |  | R.YHEEGDDAEEGEIAGGEGDGNVDESSK.S | U | 152 |
|  |  | K.SVVTEEAKPNSIENPIDRYHEEGDDAEEGEIAGGEGDGNVDESSK.S | U | 51 |
| 2 | AteIF4E1 | K.WTMTFPK.E | U | 29 |
|  |  | K.WEDPICANGGK.W |  | 44 |
|  |  | [K.NASNEAAQVSIGK.Q](http://peptide_view.pl/?file=E%3A%2FMASCOT%2Fdata%2F20170327%2FF109159.dat&query=9603&hit=1&index=O23252&db_idx=1&px=1&section=5&ave_thresh=30&_ignoreionsscorebelow=-1&report=0&_sigthreshold=0.05&_msresflags=1097&_msresflags2=258&percolate=-1&percolate_rt=0&_minpeplen=5&sessionID=all_secdisabledsession) | U | 131 |
|  |  | K.LAHGADFYCFK.H | U | 69 |
|  |  | K.SVVTEEAKPNSIENPIDR.Y | U | 98 |
|  |  | [K.EFLDYNNSIGFIIHEDAK.K](http://peptide_view.pl/?file=E%3A%2FMASCOT%2Fdata%2F20170327%2FF109159.dat&query=20266&hit=1&index=O23252&db_idx=1&px=1&section=5&ave_thresh=30&_ignoreionsscorebelow=-1&report=0&_sigthreshold=0.05&_msresflags=1097&_msresflags2=258&percolate=-1&percolate_rt=0&_minpeplen=5&sessionID=all_secdisabledsession) | U | 84 |
|  |  | K.EFLDYNNSIGFIIHEDAKK.L | U | 62 |
|  |  | K.SGVPESHPLEHSWTFWFDNPAVK.S | U | 49 |
|  |  | R.YHEEGDDAEEGEIAGGEGDGNVDESSK.S | U | 150 |

**Supplementary Table 1**. Identification of eIF4E1 in the eluates obtained from two independent CERES co-immunoprecipitation analyses from seedlings expressing *pCERES:CERES-Flag*, using the anti-Flag resin. The sequence of the peptides corresponding to eIF4E1, whether these peptides are unique for eIF4E1(U) and the score of the identified peptides in each replicate are shown. No such peptides were detected in the control samples.

**Supplementary Fig. 1. RH37 displays a putative eIF4E binding site, but it does not interact efficiently with eIF4E1.** (a) RH37 structure showing the characteristic signatures. (b) Co-immunoprecipitation analysis of RH37-Flag and HA-eIF4E1 proteins expressed in *N.benthamiana* leaves. The co-immunoprecipitation analyses were carried out as described in Fig. 1 and repeated independently three times with similar results.
